# Major liver resection for hepatocellular carcinoma in the morbidly obese: A proposed strategy to improve outcome

**DOI:** 10.1186/1477-7819-6-100

**Published:** 2008-09-10

**Authors:** Omar Barakat, Mark D Skolkin, Barry D Toombs, John H Fischer, Claire F Ozaki, R Patrick Wood

**Affiliations:** 1 Department of Surgery, the Texas Heart Institute at St. Luke's Episcopal Hospital, Houston, Texas, USA; 2 Department of Interventional Radiology, The Texas Heart Institute at St. Luke's Episcopal Hospital, Houston, Texas, USA

## Abstract

**Background:**

Morbid obesity strongly predicts morbidity and mortality in surgical patients. However, obesity's impact on outcome after major liver resection is unknown.

**Case presentation:**

We describe the management of a large hepatocellular carcinoma in a morbidly obese patient (body mass index >50 kg/m^2^). Additionally, we propose a strategy for reducing postoperative complications and improving outcome after major liver resection.

**Conclusion:**

To our knowledge, this is the first report of major liver resection in a morbidly obese patient with hepatocellular carcinoma. The approach we used could make this operation nearly as safe in obese patients as it is in their normal-weight counterparts.

## Background

Obesity is perhaps the most significant public health problem facing the United States and the Western world today. Each year, an estimated 300,000 Americans die from obesity-related illnesses [1]. The latest National Health and Nutrition Examination data show that the prevalence of obesity with body mass index (BMI) ≥ 30 kg/m^2 ^has increased from 22.9% in 1994 to 30.5% in 2000. The prevalence of morbid obesity (BMI ≥ 40 kg/m^2^) also significantly increased, from 2.9% to 4.7% [2]. This increase has affected most surgical practices, as surgeons are operating on obese patients in increasing numbers [3,4].

Perioperative morbidity, mortality, and prolonged hospital stays are particularly common in obese patients, because these patients often have preexisting cardiac and respiratory disease [3,5]. Moreover, epidemiologic studies have shown that obesity and diabetes are frequently associated with nonalcoholic fatty liver disease, which includes a spectrum of liver disorders that may progress to hepatocellular carcinoma (HCC) [6,7]. Although several studies have analyzed the impact of obesity on patients after major surgical procedures, including liver transplantation [4,8,9], there are, to our knowledge, no data on the outcome of major liver resection for HCC in morbidly obese patients.

In this report, we discuss the treatment of a large HCC in a morbidly obese patient with a BMI greater than 50 kg/m^2^. We also discuss the current literature on surgical complications in obese patients, and we make some general recommendations about treating HCC in such patients.

## Case presentation

**Figure 1:**
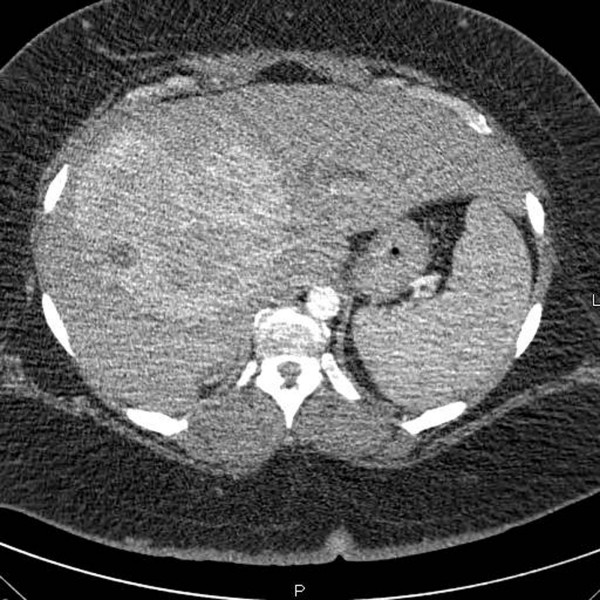
A triple-phase helical CT scan shows a 14-cm hypervascular mass involving the right lobe and the medial segment of the left lobe of the liver.

A 41-year-old woman presented with a 2-month history of pruritus. Her medical history included morbid obesity (BMI, 56 kg/m^2^), hypertension, and type II diabetes. Her initial liver function tests showed moderately elevated total bilirubin and alkaline phosphatase levels and a normal alpha-fetoprotein (AFP) level (Table 1). A computed tomography scan (CT-scan) revealed a large (14-cm), hypervascular mass that involved segment IV of the left lobe and segments V and VIII of the right lobe of the liver, partially occluding the proximal part of the common bile duct and causing moderate dilatation of the intrahepatic biliary system (Figure 1). Percutaneous biopsy of the tumor confirmed well-differentiated HCC. In addition, biopsy of segment II of the left lobe revealed mild hepatitis with no evidence of steatosis. Volumetric measurement showed that segments I, II, and III accounted for less than 20% of the total liver volume and less than 0.45% of the patient's total body weight.

**Table 1:** Preoperative liver function tests and alpha-fetoprotein (AFP) level

Total bilirubin (mg/dL)	1.8
Alkaline phosphatase (IU/L)	280
Alanine aminotransferase, ALT (IU/L)	80
Aspartate aminotransferase, AST (IU/L)	81
Albumin (g/dL)	3.8
AFP (ng/mL)	3

Surgical resection was initially ruled out because of a small-for-size remnant liver and abnormal pulmonary function tests that suggested a combination of restrictive and peripheral airway diseases (Table 2). After discussing with the patient the risk of complications and potential liver failure associated with extensive liver resection, we elected to pursue locoregional therapy consisting of hepatic transarterial chemo/radioembolization with doxorubicin and yttrium-90 (Y-90) microspheres (Sirtex Medical Limited, Lake Forest, IL, USA). The patient was also placed on a weight-reduction program based on a hypocaloric Mediterranean diet, which has been proven effective for weight loss. Protein intake was calculated as 1 g/kg of body weight. The patient was also instructed to enroll in an aerobic and resistance exercise program in an attempt to improve her metabolic syndrome.

**Table 2:** Pulmonary function test results at initial evaluation and immediately before and after operation

Parameter	Initial value	Postoperative value
Forced vital capacity (L)	3.38	3.73
Forced expiratory volume in 1 second (L)	1.96	2.93
Maximum voluntary ventilation (L/min)	42	75
Vital capacity (L)	2.6	3.7
Total lung capacity (L)	4.1	5.9
Functional residual capacity (L)	1.6	2.3
Expiratory reserve volume (L)	0.02	0.06

The treatment protocol consisted of 6 weekly injections of doxorubicin mixed with ethiodized oil, followed by 500- to 700-micron Embospheres (Biosphere Medical Inc, Rockland, MA, USA) alternated with Y-90 microspheres injected selectively into the right and middle hepatic arteries by interventional radiologists. The patient underwent 5 cycles of treatment; side effects were minimal and were related to postembolization effects. The total cumulative doses of doxorubicin and Y-90 were 200 mg and 40.4 mCi, respectively.

After 7 months of treatment, a follow-up CT scan of the abdomen showed no significant change in the size and enhancement pattern of the tumor. However, the patient's weight had decreased from 159 kg to 136 kg (so that BMI decreased from 56 to 48 kg/m^2^). This change was accompanied by improvements in most pulmonary function parameters (Table 2) and reductions in the dosage of the patient's antihypertensive and antidiabetic medications. At that time, the decision was made to proceed with extended right hepatectomy to remove segments IV, V, VI, VII, and VIII after right portal vein embolization (PVE) to allow compensatory hypertrophy of segments II and III. A volumetric study performed 8 weeks after PVE showed that the caudate lobe and segments II and III accounted for 33% of the total liver volume.

### Surgical technique

The patient underwent an extended right hepatectomy. She was positioned on a bariatric operating table (Maquet surgical table; Getinge AB, Getinge, Sweden). Exploratory laparotomy was performed through bilateral subcostal incisions with upper midline extensions. A bariatric Thompson self-retaining retractor (Thompson Surgical Instruments, Inc., Traverse City, MI, USA) was used to elevate the costal margins and facilitate exposure. Despite extensive locoregional therapy, there was minimal inflammatory reaction and adhesions between the liver and adjacent organs. Intraoperative ultrasound was used to confirm the previously defined anatomic relation of the tumor with the intrahepatic vasculature. Hilar dissection and mobilization of the right lobe of the liver were carried out in standard fashion for extended right hepatectomy. Parenchymal transaction was performed with a dissecting sealer (TissueLink Medical, Inc., Dover, NH, USA). The total operative time was 630 min. Estimated blood loss was 720 mL. No transfusion of blood products was required.

The patient's postoperative course was uneventful, despite the long operative time and the technical difficulties encountered during mobilization of the liver because of the compensatory hypertrophy of the left lateral segment and the tumor's large size. The patient remained in the intensive care unit for 2 days and was discharged from the hospital on postoperative day 6. However, superficial wound dehiscence developed that involved the skin and the subcutaneous tissue. This was treated with vacuum-assisted closure (with the VAC Therapy system; KCI, Inc, San Antonio, TX, USA), which facilitated wound healing by secondary intention in 8 weeks.

Histopathologic examination of the excised tumor and portion of the normal liver revealed a well-differentiated 11-cm HCC. There were focal areas of necrosis and hemorrhage from previous chemoradiation therapy, but there was no evidence of microvascular invasion. In the normal liver parenchyma, there was evidence of postembolization effects, mainly focal areas of foreign body giant cell reaction, but minimal fibrosis and no steatosis. All lymph nodes were negative for malignancy. Currently, the patient is doing well, with no evidence of recurrence 17 months after tumor resection.

## Discussion

Several studies have found that obesity increases the risk of complications and length of hospital stay and is independently associated with increased mortality after elective abdominal surgery [10-13]. In contrast, a prospective study of 6336 patients who underwent elective noncardiac surgery at a university hospital found that obesity alone was not a risk factor for postoperative complications [14,15]. However, these findings were probably due to the unusually low prevalence of major comorbidities in the obese patients in these studies.

In a large study of 18,172 adult patients, including 3877 obese patients, who underwent LT in the US between 1988 and 1996, the rates of primary graft nonfunction and of 1- and 2-year mortality were significantly higher in the morbidly obese patients than in the other patients. The authors of that study recommended that morbid obesity (BMI > 35 kg/m^2^) be considered a relative contraindication for LT [16].

With regard to our morbidly obese patient (BMI, 56 kg/m^2^) with a large HCC, during the initial surgical evaluation, she was considered a high-risk candidate for extended right hepatectomy because of her markedly abnormal pulmonary function test results and the insufficient volume of the left lateral segment of her liver. We believe that the neo-adjuvant treatment protocol we implemented prevented tumor progression during the aggressive weight-reduction program that the patient was instructed to follow. This program was instituted because pulmonary function test results and respiratory drive parameters have been found to improve markedly after weight loss [17].

The locoregional therapy protocol we implemented was chosen on the basis of evidence that combination therapy achieves a higher response rate than repeated TACE alone in large HCCs [18,19]. Yttrium-90 microsphere injection is a novel form of transarterial radiotherapy that has been used increasingly for HCC as a single agent, and it has produced a good response rate [20,21]. To our knowledge, no study has evaluated the use of radioembolization in conjunction with other treatment modalities for any type of malignant disease. However, evidence suggests that doxorubicin hinders the repair of radiation-induced DNA damage in HCC; thus, these treatments may have a synergistic therapeutic effect [22].

As we anticipated, the tumor was found to be receiving its blood supply from both branches of the hepatic artery. To prevent ischemic injury to segments II and III of the left lobe, we avoided injecting the embolization particles through the left hepatic artery that supplied the lateral aspect of the tumor. This might explain the tumor's failure to respond despite repeated treatments. On the other hand, selective injection into the middle and right hepatic arteries might have spared segments I, II, and III the adverse effects of chemoradiation treatment that were seen in non-tumorous segments of the right lobe.

Preoperative portal vein embolization is becoming a standard technique for inducing compensatory hypertrophy of the remaining liver and improving the safety and rate of resectability in patients with small-for-size remnant livers [23,24]. Furthermore, sequential preoperative arterial and portal venous embolization can induce tumor necrosis and hypertrophy of the normal liver, which allow safe resection and longer recurrence-free survival [25,26].

We would have continued the locoregional therapy had there been evidence of tumor response. On the other hand, if the tumor had progressed, we would have added systemic therapy, such as administering the multikinase inhibitor sorafenib, to the treatment protocol. The decision to proceed with surgical resection was based on the tumor's lack of response and, more importantly, on the improved pulmonary function and reduced metabolic syndrome that resulted from the successful weight-reduction program the patient followed during locoregional treatment.

## Conclusion

To reduce the risks that major liver resection poses in morbidly obese patients with significant comorbidity, we suggest implementing a dietary weight-reduction and exercise program to improve the performance status of these patients before resection. While this program is underway, regional therapy can be implemented to prevent the tumor from progressing to the point of inoperability. Portal vein embolization may be required before resection to increase the volume of the remnant liver and to reduce the risk of liver failure and other postoperative complications. We believe that further studies that include large numbers of patients are needed to determine the upper limit of BMI for performing extensive liver resection safely in morbidly obese patients.

## List of abbreviations

AFP: Alpha-Fetoprotein; BMI: Body Mass Index; CT: Computed Tomography; HCC: Hepatocellular Carcinoma; LT: Liver Transplantation; PVE: Portal Vein Embolization.

## Competing interests

The authors declare that they have no competing interests.

## Authors' contributions

OB: Performed the operation, devised the therapeutic plan, and wrote the manuscript. MS: Performed the TACE; helped in drafting the manuscript. BT: Performed the TACE and Y-90 Sir-Sphere treatment, and helped in drafting the manuscript. JF: Performed the portal vein embolization and TACE, and helped in drafting the manuscript. CFO: Helped in drafting the manuscript. RPW: Co-surgeon during the operation; helped in designing the therapeutic plan, and proofread the manuscript.

## Consent

Written informed consent was obtained from the patient for publication of this case report and any accompanying images. A copy of the written consent is available for review by the Editor-in-Chief of this journal.
